# A Rare Case of Severe Isolated Right Heart Failure with Secundum Type Atrial Septal Defect and Mitral Regurgitation Without Pulmonary Hypertension

**DOI:** 10.7759/cureus.34112

**Published:** 2023-01-23

**Authors:** Muhammad S Sabri, Faiza Riaz, Nain Tara, Maheen Anwar, Muhammad Usman Khan

**Affiliations:** 1 Department of Cardiology, Hospital Corporation of America Houston Healthcare Northwest, Houston, USA; 2 Department of Paediatrics, Combined Military Hospital, Lahore, PAK; 3 Department of Medicine, AIlama Iqbal Medical College, Lahore, PAK

**Keywords:** pulmonary hypertension, pericardial effusion, left heart failure (lhf), mitral regurgitation (mr), secundum type atrial septal defect (asd), lutembacher syndrome (ls)

## Abstract

Typically, right heart failure (RHF) may occur following left heart failure (LHF) in chronic volume overload states such as chronic severe mitral regurgitation (MR) through chronically elevated pulmonary pressures. In Lutembacher syndrome (LS), the direct shunting through a secundum type atrial septal defect (ASD) results in congestive heart failure in the setting of severe mitral stenosis (MS) with or without elevated pulmonary arterial or venous pressures. We report a rare case of severe isolated RHF and bi-atrial enlargement resulting from the direct shunting through a secundum type ASD in the presence of a severe eccentric primary MR. There are no significant cases documented like this after a thorough search using PubMed, Medline, and Google Scholar. A review of the literature suggests that LS is also caused by a combination of mitral regurgitation and a secundum-type atrial septal defect without mitral stenosis, though rarely. Because this is a primary MR, we feel it is a case of LS with MR, ruling out a combination of secondary MR and secundum-type atrial septal defect.

## Introduction

Lutembacher syndrome (LS) is defined as the combination of the congenital atrial septal defect (ASD) with acquired or congenital mitral stenosis (MS) [1]. However, few authors also allocate ASD with mitral regurgitation (MR) as a part of the LS spectrum [2]. Typically, the association between septum secundum ASD and MR is characterized by mitral valve abnormalities and pulmonary arterial hypertension (PAH) [3]. But we present a rare presentation of suspected LS with MR, which has isolated right heart failure (RHF) from the hemodynamic interplay between an MR and co-existent large ASD (22 mm) without PAH or left ventricle (LV) dysfunction. The natural history of LS depends on the size of ASD, the severity of mitral valve pathology, and compliance of the right ventricle (RV) [4].

## Case presentation

A 68-year-old female, with a past medical history of hypertension, cardiomegaly, atrial fibrillation, and hypothyroidism, presented to the emergency department (ED) for worsening symptoms of lower extremity edema, shortness of breath, and poor exercise tolerance for four weeks. Her temperature was 98.4 F, heart rate was 141 beats/min (bpm) with an irregularly irregular pulse, respiratory rate was 18 breaths/min, and blood pressure was 116/82 mmHg. Physical examination revealed an irregularly irregular pulse with a pulse deficit of about 12 beats/min, distant heart sounds, reduced breath sounds at lung bases, and jugular venous pressure of approximately 5 cm H_2_O.

**Figure 1:**
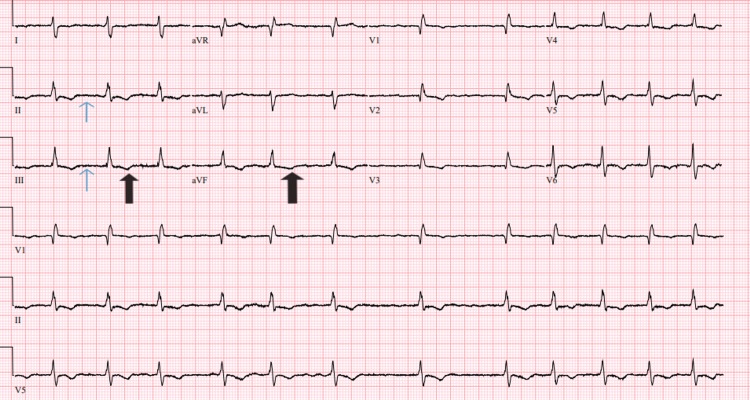
Electrocardiogram The image shows atrial fibrillation (blue arrows), and abnormal T waves (black arrows).

Complete blood counts, basic metabolic profile, thyroid function tests, and iron studies were within normal limits except for sodium which was 132mEq/L. Electrocardiogram (ECG) showed atrial fibrillation with a ventricular rate of 73 bpm, QRS duration 117 milliseconds (ms), corrected QT (QTc) 430 ms, low voltage complexes, marked right axis deviation, right bundle branch block (RBBB), and T wave abnormalities in anteroseptal and inferior leads (Figure 1). Computed tomography angiography (CTA) chest was unremarkable for pulmonary embolism (PE) or any appreciable pulmonary parenchymal or vascular disease. She was admitted and started on intravenous (IV) diuresis with furosemide (40 mg IV q8 {every 8 hours}), metoprolol tartrate (12.5 mg PO {orally} q12 {every 12 hours}), and enoxaparin (30 mg SQ {subcutaneous} q12).

**Figure 2:**
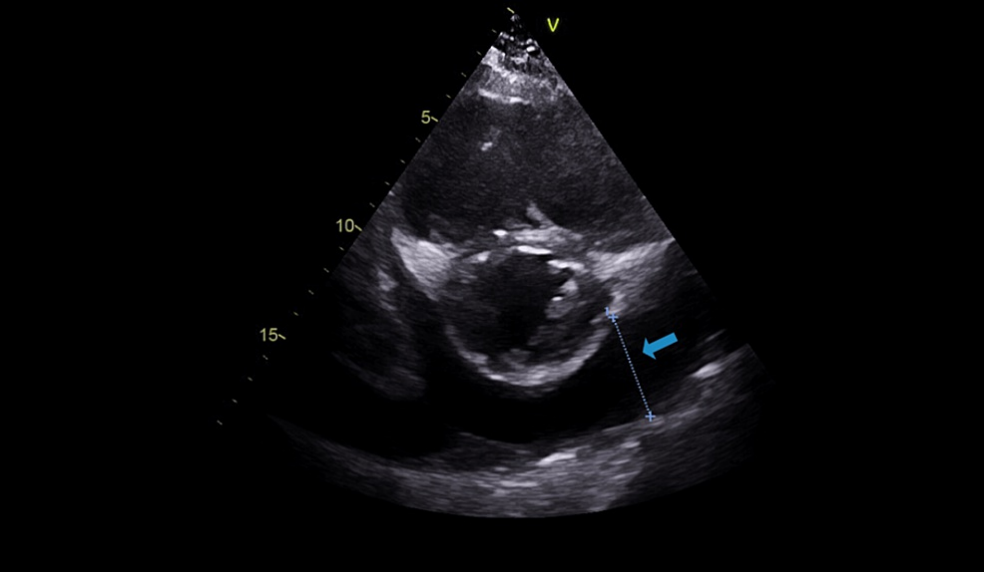
Echocardiogram (image 1) The blue arrow shows pericardial effusion.

**Figure 3:**
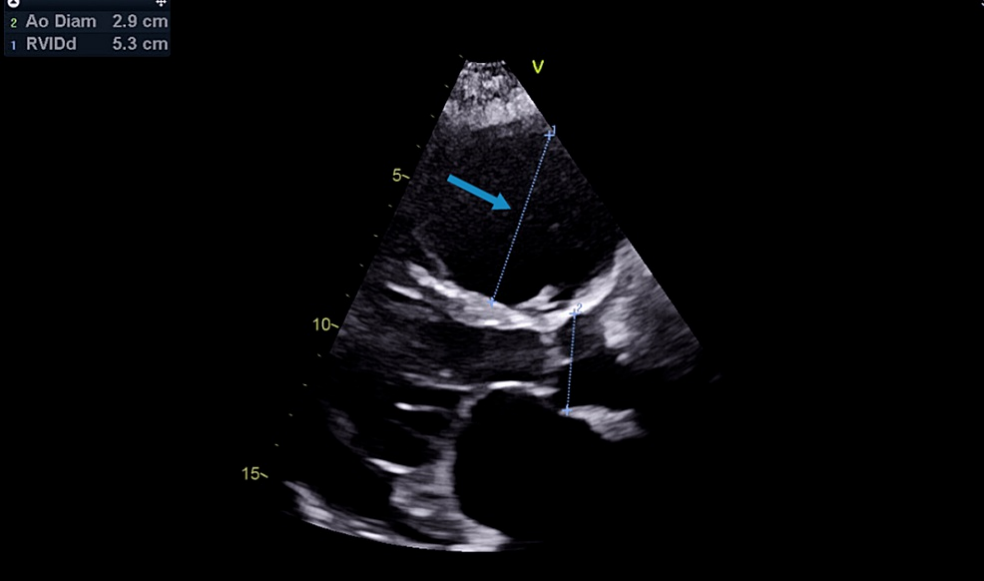
Echocardiogram (image 2) The blue arrow shows dilated right ventricle (RV).

**Figure 4:**
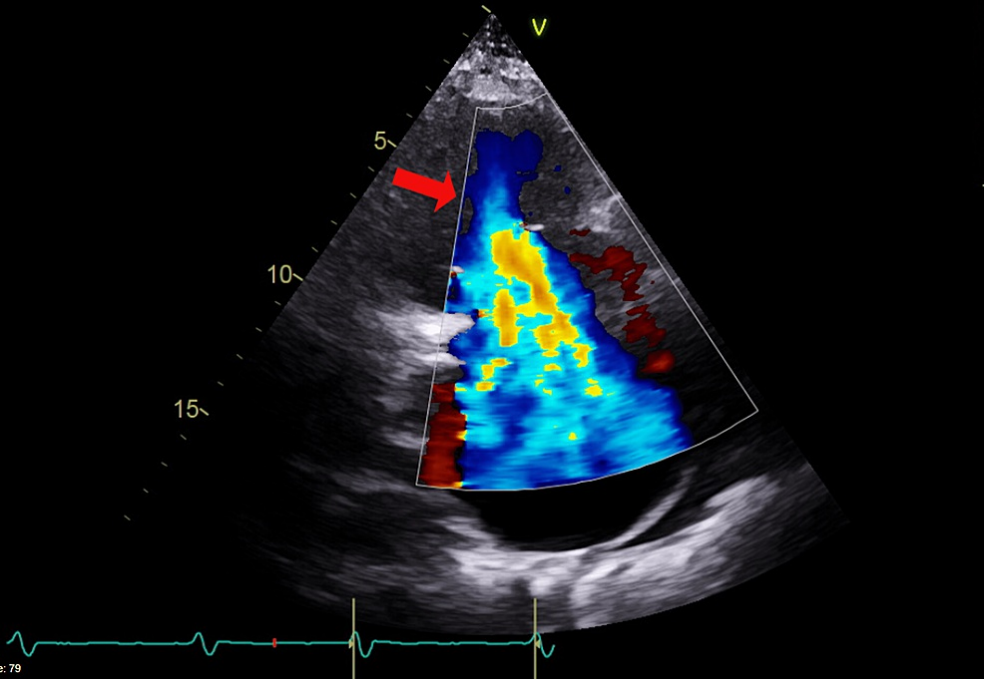
Echocardiogram (image 3) The image is showing tricuspid regurgitation (TR) (red arrow).

A subsequent echocardiogram showed a circumferential moderate to large-sized pericardial effusion (2.2 cm (Figure 2), severe bi-atrial enlargement, severely dilated (Figure 3) and dysfunctional RV with an end-diastolic diameter of 5.3 cm, severe central tricuspid regurgitation (TR) (Figure 4) with normal appearing leaflets and severe eccentric posteriorly directed MR. LV size, systolic function (ejection fraction 55-60%), wall thickness, and motion were within normal limits.

A right heart catheterization performed later showed an uptrend of oxygen saturation from 72.8% in the superior vena cava (SVC) to 85.9% in the right atrium with normal pulmonary arterial pressure (PAP) and pulmonary vascular resistance 35.5 dynes/sec/cm^-5^,^ ^pulmonary capillary wedge pressure (PCWP) of 13 mmHg, pulmonary artery (PA) pressure of 34/8 (mean of 17 mmHg), RV end-diastolic pressure of 4 mmHg, right atrial (RA) pressure of 5 mmHg. The Qp: Qs ratio of 3.1 showed a significant left-to-right shunt. The Nyquist limit was 40cm/sec. Oxygen saturations included PCWP 86.3%, PA 85.4%, RV 83.7%, and RA 85.9%. SVC 72.8%, inferior vena cava (IVC) 81.7%, and systemic arterial 94%. A left heart catheterization showed normal LV end-diastolic pressure (11 mmHg). The coronary angiography revealed no significant coronary artery disease (CAD). 

The patient had adequate heart rate control with metoprolol. Her symptoms gradually improved with cautious diuresis and strict input-output monitoring. Subsequent trans-esophageal echocardiography (TEE) confirmed a medium-sized, 2.2 cm secundum type ASD with the left to right shunt (Video 1), severe primary mitral valve regurgitation (Video 2), with an eccentric posteriorly directed jet due to underlying posterior leaflet restriction. 

**Video 1:** TEE showing blood flow across the ASD TEE: Trans-esophageal echocardiography; ASD: Atrial septal defect

**Video 2:** TEE showing MR TEE: Trans-esophageal echocardiography; MR: Mitral regurgitation

The patient underwent mitral valve replacement with predominant chordal sparing (only A2 resected), with 29 mm Medtronic Mosaic stented porcine xeno prosthesis (Medtronic, Houston, Texas), closure of ostium secundum ASD via bovine pericardial patch plasty (Edwards, Houston, Texas), tricuspid valve repair via De Vega suture annuloplasty and exclusion of left atrial appendage with 40 mm Atricure Atriclip (Atricure, Houston, Texas). Pericardial effusion was drained during the operation, and laboratory analysis confirmed a transudative etiology. Gram stain, bacterial and fungal cultures were negative. Cytology was unremarkable. Acid-fast bacillus (AFB) stain and mycobacterial culture along with adenosine deaminase were negative.

The postoperative period was unremarkable. Later after stabilization, the patient was discharged on metoprolol, oral furosemide, and warfarin. She was regularly seen in outpatient. At a two-month follow up she reported the resolution of her symptoms. A physical exam revealed no signs of RHF and the echocardiogram revealed normal functioning mitral valve prosthesis, no pericardial effusion, marked reduction in RV size (3.8 cm), and improvement in RV systolic function.

## Discussion

RHF causes can be classified into three types: due to pulmonary hypertension, RV and tricuspid valve abnormalities, and pericardial disorders [5]. The leading cause of RHF is LHF through resultant pulmonary congestion and pulmonary hypertension [5]. LHF, whether caused by systolic heart failure (HF), HF with maintained LV systolic function, or severe mitral valve disease, causes pulmonary hypertension (PH) and, if untreated, leads to RHF [5]. The most frequent causes of MR include mitral prolapse, leaflet pathology, rheumatic heart disease (RHD), infective endocarditis (IE), CAD, drugs (ergotamine, methysergide), and collagen vascular diseases [6]. The decompensated chronic MR leads to increase left atrium (LA) pressures that are transmitted back to the pulmonary vasculature. Initially, it is reversible and manifests as pulmonary edema. Chronically remodeling in the pulmonary vessels leads to irreversible pulmonary hypertension. Earlier in the course, chronic severe MR leads to compensatory LV and LA dilation. But with time, the untreated left ventricle fails and leads to decompensated LHF and eventually RHF.

Isolated RHF can be due to pulmonary causes (pulmonary hypertension), breathing disorders (sleep apnea), abnormalities of tricuspid and pulmonary valve disease, chronic pulmonary embolism, or infarction of the right side of the heart [5]. The patient usually becomes symptomatic in the third to the fourth decade [7]. We are presenting a rare case of isolated RHF in the setting of septum secundum defect and severe eccentric MR with normal LV function and pulmonary circulation pressures.

The pathophysiology of this case appears to be the outcome of MR and ASD, with no involvement of the LV or pulmonary vasculature. It is plausible that the regurgitated blood through the mitral valve (MV) is circulated in a loop involving the LA to RA through the ASD, followed by emptying into the RV, thus causing dilated RV cardiomyopathy and secondary TR [8]. MR, in this case, did not lead to a backup of blood to pulmonary vessels rather it was shunted across ASD. The preferential shunting of blood to RA via ASD is substantiated by the pathway of least resistance and lowest pressures through a significant-sized ASD [8] (2.2cm in the patient) given the compliant [9] nature of the right side of the heart. Thus, this phenomenon prevents pulmonary vascular changes to the detriment of RV and gives the clinical picture of isolated RHF. Atrial dilatation caused by the volume overload mechanism may contribute to atrial fibrillation [8], as seen in this patient.

With regards to the etiology of primary MR as in this patient, it is important to note that elder people with uncorrected septum secundum usually present with symptoms of pulmonary congestion [10]. Among people with ASD, the structural cause of MR was found to be fibrosis of the leaflet and chordae tendinae [11]. Many septum secundum cases are documented as having mitral valve changes secondary to increased blood flow, abnormal cusp movement, and resultant valvular damage causing fibrosis of the valve [12]. Septum secundum ASD has also been found in association with posterior leaflet mitral valve prolapse that may have or not have clinical evidence of mitral valve lesion and MR [13]. Usually, people with mitral valve regurgitation caused by large septum secundum ASD develop pulmonary hypertension due to increased blood flow in the pulmonary circulation [14]. 

People with LS can develop RHF with even moderate pulmonary hypertension due to the shunting of blood flow through the ASD [15]. Moderate pulmonary hypertension develops due to excessive blood coming in the right side of the heart by shunting via ASD and then passing through the pulmonary vessels to the LA [16]. LS is more prevalent in females. MS managed by valvulotomy can sometimes cause iatrogenic LS [17].

With regards to the pericardial effusion, a transudative pericardial effusion may be caused by heart failure, hypoalbuminemia, post-radiation therapy, and liver or renal insufficiency [18]. In our case, right ventricular dilation reflects abnormal volume/pressure relationships in the right heart, and this irregularity, via changes in venous and lymphatic drainage, supports the buildup of pericardial effusion in these individuals with heart disease, with or without congestive heart failure [19]. 

This case report uniquely highlights the hemodynamic interplay of severe primary MR and secundum type ASD resulting in severe RV failure that correlates with LS with MR, a very rare type of LS.

## Conclusions

Aside from several causes of isolated RHF, one of the etiologies of isolated RHF stems from chronic eccentric primary MR along with secundum ASD with no diagnostically proven change in PAP and LV function. Previously many cases have been studied that revolve around MS causing RHF through ASD with or without elevated PAP and LV failure, which is a typical occurrence of LS. Therefore, our case is a rare and atypical report of LS that would add more to the literature by guiding physicians that LS can be demonstrated with MR at any age and female preponderance while making a diagnosis. This case is in parallel but still different from typical LS. And the different pathophysiology in this emanates from the fact that an increase in left atrial volume by MR is channeled to the right atria across significant-size ASD instead of back pooling pressures in the pulmonary vasculature raising RA volume and subsequently RHF.
